# The impact of intergenerational programs on social capital in Japan: a randomized population-based cross-sectional study

**DOI:** 10.1186/s12889-019-6480-3

**Published:** 2019-02-06

**Authors:** Yoh Murayama, Hiroshi Murayama, Masami Hasebe, Jun Yamaguchi, Yoshinori Fujiwara

**Affiliations:** 1 Research Team for Social Participation and Community Health, Tokyo Metropolitan Institute of Gerontology, 35-2 Sakae-cho, Itabashi-ku, Tokyo, 178-0015 Japan; 2 Institute of Gerontology, The University of Tokyo, Tokyo, Japan; 3 Department of Human Welfare, Seigakuin University, Saitama, Japan

**Keywords:** Intergenerational program, Social capital, Senior volunteer aging

## Abstract

**Background:**

Over the last several decades, social isolation and loneliness among older adults have posed an increasingly urgent challenge due to the rapidly aging population in Japan. To remedy the situation, many communities have introduced intergenerational programs. However, few studies have investigated the benefits of social capital across generations as a result of intergenerational interaction between children and older generations. Therefore, we aim to ascertain the degree to which intergenerational programs that take root in a community will affect the social capital of all generations in the community.

**Methods:**

We focus our research on one specific program, REPRINTS, an intergenerational health promotion program for older adults that has been active for over 10 years in Tama Ward, Kawasaki City, Kanagawa Prefecture. We conducted a population-based cross-sectional study of residents between the ages of 20 and 84 years who were randomly selected from the basic resident register. Approximately 2500 residents were selected, of which 978 responded; data from 891 respondents were analyzed.

**Results:**

Hierarchical linear modeling suggests that the duration of programs was a significant community-level indicator of neighborhood trust. At the individual level, people between 30 and 59 years of age and people over 60 years of age have more positive effects on neighborhood trust than do people between 20 and 39 years of age.

**Conclusions:**

The ongoing intergenerational programs between older citizens and children can enforce neighborhood trust, thus strengthening a community’s intergenerational ties. The REPRINTS program has been developed through cooperation with local citizens, senior volunteers, and teachers from the community. Its collaborative nature ensures longevity and continuous growth in a community. It is challenging to create long-term intergenerational programs that take root in communities, making persistence and collaboration a crucial factor in fruitful intergenerational relationships. Overall, ongoing intergenerational program implementation associates with building social capital, thereby strengthening potential intergenerational ties and promote mutual support among local residents which will reduce or prevent social isolation among older.

## Background

Over the past few decades, the population of several developed countries, including Japan, has begun to age rapidly. At the same time, studies show that the number of older people who live alone is increasing and that older citizens are more socially isolated than they have ever been [1, 2]. Other research suggests that social isolation and loneliness have negative effects on physical and mental health [3]. As a result of these findings, increasing attention is being placed on helping older people maintain interpersonal relationships. One possible way to build interpersonal relations is to develop greater social capital within local communities [4]. *Social capital* is defined as social networks that share norms, values, and understandings, facilitating cooperation within or among group [5, 6]. Previous studies have shown that social capital has a positive effect on several health outcomes, including mortality, hospitalization, self-rated health, and depression [7–9].

To network local residents and reconstruct communities, many local communities have implemented intergenerational programs [10–15]. An *intergenerational program* is a social service that involves the ongoing and purposeful exchange of resources between members of younger and older generations [16, 17]. Intergenerational programs were started in the United States to address emerging social problems, including improving educational success for young people, reducing ageism, and increasing the quality of life for older citizens [18–21]. For example, Experience Corps® is an intergenerational program that began in the United States in 1996. It trains senior volunteers to work in schools to improve students’ academic outcomes, and Experience Corps® volunteers assist teachers with literacy and library work [22].

Previous studies have shown that intergenerational programs have positive influences on several outcomes. Older adults benefit from improved physical and mental health, and increased social activities [10, 22–29]. School children will benefit from these programs through improved academic performance, positive perception of the elderly, and attitudes toward community activities [30–32]. Moreover, intergenerational programs would develop social relationships across generations through cooperation with local coordinators, senior volunteers, and teachers from the community. This would further strengthen potential intergenerational ties and promote mutual supports among local residents [25, 33].

**Fig. 1:**
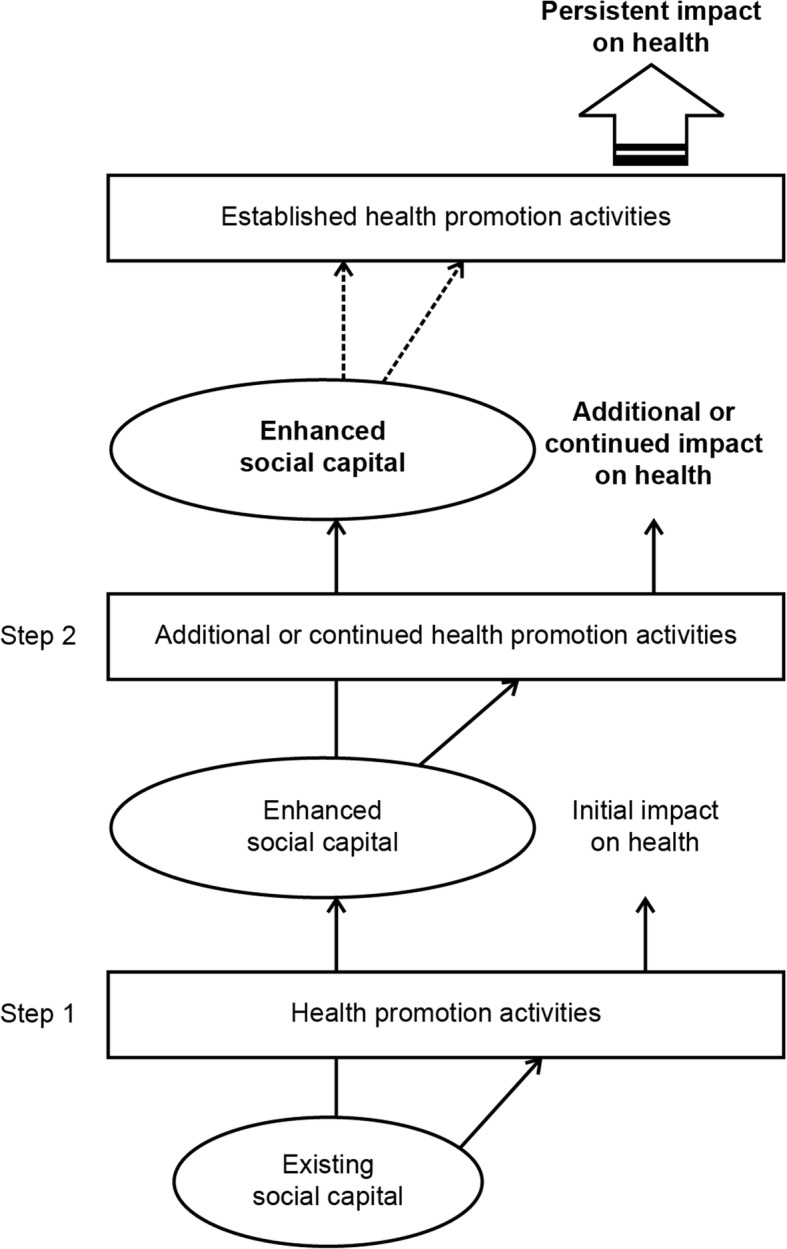
Illustration of the desired relationship between social capital and health promotion intervention programs [12]

While these studies have focused on understanding the individual benefits of intergenerational programs, few studies have investigated the effects on social capital. One exception is the work of Bostrom [33], who conducted a questionnaire-based survey among participants in the “Class Granddad” program run in a limited number of Stockholm schools. Demonstrating that the program has an effect on pupils’ cognitive social capital—that is, the individual’s subjective perception of social resources —Bostrom [33] indicates that the social capital benefits of intergenerational programs spill over to other generations as a result of intergenerational interaction (Fig. 1). Figure 1 illustrates the relationship between social capital in the community and health promotion intervention programs, proposing that enhanced social capital can positively influence the continuation and impact of such programs. In other words, this model indicates that the social capital benefits of one generation spill over to other generations over time. Similarly, Bekkers et al. [34] have suggested that the intensity and duration of contact can promote the trust. This suggests that ongoing intergenerational programs increase the social capital of not only the target age group but all the generations in a community. However, few studies have investigated the effects of spillover on social capital in local communities. In order to verify the effects of spillover on social capital, we aim to investigate the association between the degree to which intergenerational programs take root in the community and social capital among local residents. We then focus on the REPRINTS program, an intergenerational program training senior volunteers to work in schools.

The “Research of Productivity by Intergenerational Sympathy,” or REPRINTS, program in Japan, trains volunteers over the age of 60 to read picture books to school children [35]. The program started in three areas: Chuo Ward, central Tokyo; Tama Ward, Kawasaki City, Kanagawa Prefecture, a suburb of Tokyo; and Nagahama City, Shiga Prefecture, a rural area in western Japan. REPRINTS participants were recruited from March to July 2004 through community newspapers, newsletters, and events advertising the program. After submitting a volunteer application, applicants attended intensive weekly training seminars for three months where they underwent projects involving picture book reading. Thereafter, they began reading picture books in elementary schools, kindergartens, and public childcare centers. Volunteers were asked to participate once every one to two weeks in groups of about six to ten members. By 2016, the number of locations had increased to eight and the number of senior volunteers had reached about 300 people [36].

Thus, we hypothesized that the degree to which REPRINTS programs take root in a community will affect the social capital of all generations in the community.

## Methods

### Participants

We focused our research on the REPRINTS program in Tama Ward, Kawasaki City, which has a total population of 214,158 residents (about 104,099 of which are female) as of October 2015 [37]. The program had been around for approximately ten years at the time of our research. In 2016, 65 senior volunteers in Tama participated in about 30 institutions, including schools, kindergartens, and public childcare centers.

We conducted a survey among local residents, randomly selecting 2500 (of 171,167) respondents between the ages of 20 and 84 from the basic resident register system—the official Japanese system of recording residents—in Tama ward, using a stratified random sampling method by age and gender. The selected residents received the questionnaire in March 2015, and 978 residents (461 male and 517 female) responded, resulting in a response rate of 39.1%. Response rates varied across the districts: SD = 5.75; Range, 28.3–50.5%. We excluded responses from respondents with missing values for key variables. After eliminating all missing data, we analyzed the data of 891 residents in total.

### Outcome measures

We asked respondents for the following demographic information: age in years, gender, highest school grade achieved, living arrangement, duration of residence, and perceived financial status.

We assessed social capital through two question items, both used in previous studies on social capital [38–41]. First, we asked respondents to rate whether they trusted their neighborhood (neighborhood trust, “people in this neighborhood can be trusted”) using a 5-point Likert scale from 1 (agree) to 5 (disagree). Second, we asked respondents whether they want to be helpful in their neighborhood (neighborhood norms, “people in the neighborhood are willing to help one another”) using a 5-point Likert scale ranging from 1 (agree) to 5 (disagree).

We measured the degree to which the REPRINTS programs take root in the community by the program’s duration; how long the program had been around, number of activities, and recognition of the program. Some of this information was obtained from program staff. Table 1 shows that REPRINTS spread gradually throughout Tama over a period of 11 years. By the time of our study, it was active in 13 of 24 districts (Duration of program: Mean = 1.37, *SD* = 1.09, Number of programs: Mean = 1.05, *SD* = 0.97). There was no difference in response rate between REPRINTS program intervention districts and non-intervention districts: *t*(22) = 0.71, *p* = 0.48).

**Table 1:** REPRINTS’ degree of penetration in Tama Ward districts

Duration	2004–2005	2006–2010	2011–2015	No intervention
	(Term 1)	(Term 2)	(Term 3)	
	3	7	3	11
Number	One place	Two places	Three places	No intervention
	(Type 1)	(Type 2)	(Type 3)	
	9	2	2	11

Note. The numbers in the table represent the number of Tama ward districts

We categorized the duration of REPRINTS programs by breaking them into four categories based on when they began: Term 1 (2004–2005), Term 2 (2006–2010), Term 3 (2011–2015), and districts with no intervention programs. We then quantified the number of activities as the number of types of institutions in each district where senior volunteers participated: Type 1 (one institution: kindergarten, elementary school, junior high school, etc.), Type 2 (two institutions), Type 3 (three institutions), and districts with no REPRINTS intervention. Finally, we assessed the recognition of REPRINTS through the question, “Do you know whether the REPRINTS, senior volunteers for picture book reading to children, has worked in Tama Ward?” answered according to a 3-point Likert scale ranging from 1 (“I don’t know about them at all”) to 3 (“I know about REPRINTS”). We then asked the respondents who claimed to know about REPRINTS whether they, their family members, or acquaintances have been participated in the REPRINTS program.

### Data analysis

Our descriptive data analysis used chi-square tests or a one-way ANOVA test to compare demographics and basic variables among age groups in IBM SPSS version 20.0. As local residents are nested within local communities, we examined relationships across these levels using hierarchical linear modeling in HLM 7.02, with neighborhood trust and neighborhood norms as the outcome [42]. We constructed three models and used deviance as a global adjustment measure. The first model (Model 1) was examined to calculate the intraclass correlation coefficient in order to estimate how much of the total variance in neighborhood trust or neighborhood norm was associated with community context. In Model 2, the individual-level variables (e.g., gender, recognition of intergenerational programs, duration of residence, and perceived financial status) were inserted. Model 3 added the community-level variables, including the duration and number of intergenerational programs.

## Results

Table 2 shows the demographic results of our survey. The mean age was 49.5 (range: 20–83). The sample was predominantly comprised of females (53.0%) with at least a high school education (66.8%). The majority of the participants lived with others (85.2%), while 13.3% lived alone. According to the 2015 population census, 34.6% of the general population lived alone in 2015 [43], indicating that the ratio of our sample is higher than that of the general population. We also found a statistically significant difference in the duration of residence and the perceived financial status among different age groups. Residual analysis showed that people over the age of 60 lived in their communities longer than people in their 20s and 30s. There were significant differences in the scores for neighborhood norms and neighborhood trust among different age groups. Bonferroni’s multiple comparison tests showed that the scores for people over the age of 60 and between the ages of 40 and 59 were significantly higher than that for people between the ages of 20 and 39 years. There were no differences between the age groups with regard to the recognition of the program. Although there were no REPRINTS participants among respondents, eight respondents claimed that family members or acquaintances participated in REPRINTS programs.

**Table 2:** Demographic results

Individual characteristics	All	20–39 years (*n* = 285)	40–59 years (*n* = 316)	60+ years (*n* = 290)	*p*-value
	*n* (%) or M (SD)	*n* (%) or M (SD)	*n* (%) or M (SD)	*n* (%) or M (SD)	
Gender					
Male	419 (47.0)	135 (47.4)	146 (46.2)	138 (47.6)	0.934
Female	472 (53.0)	150 (52.6)	170 (53.8)	152 (52.4)	
Education					
Less than high school	58 (6.5)	2 (0.7)	11 (3.5)	45 (15.5)	0.001
High school degree	238 (26.7)	59 (20.7)	71 (22.5)	108 (37.2)	
More than high school	595 (66.8)	224 (78.6)	234 (74.1)	138 (47.6)	
Living arrangement					
Lives alone	119 (13.3)	43 (15.2)	35 (11.1)	41 (14.7)	0.270
Lives with others	763 (85.2)	240 (84.8)	281 (88.9)	238 (85.3)	
No Answer	14 (1.6)	2 (0.7)	0 (0.0)	11 (3.8)	
Duration of residence					
Over ten years	509 (57.1)	211 (26.0)	136 (57.0)	35 (87.9)	0.001
Perceived financial status					
Slightly or Extremely	331 (37.1)	119 (42.8)	103 (32.6)	109 (37.6)	0.067
Sufficient Social capital					
Neighborhood trust	3.29 (0.91)	3.09 (0.97)	3.29 (0.86)	3.47 (0.86)	0.001
Neighborhood norm	3.21 (0.89)	3.06 (0.88)	3.26 (0.87)	3.31 (0.90)	0.002
Recognition of program	1.19 (0.51)	1.14 (0.49)	1.22 (0.54)	1.19 (0.48)	0.191

Notes: *p* values obtained using chi-square test or one-way ANOVA test

Tables 3 and 4 show the relationship between the degree to which the REPRINTS programs take root in a community, neighborhood trust, and neighborhood norm for each age category. With regard to neighborhood trust, the duration of programs was a significant community-level indicator of neighborhood trust. At the individual level, the duration of residence and living alone was negatively associated with neighborhood trust, while perceived financial status and recognition of program was positively associated with neighborhood trust. Furthermore, people between the ages of 30 and 59 and those over the age of 60 were more positively associated with neighborhood trust than people between the ages of 20 and 39. The fit of model 3 proved better than that of the previous model.

**Table 3:** Multilevel models predicting neighborhood trust

	Model 1			Model 2			Model 3		
	Coeff.	SE	*p-*value	Coeff.	SE	*p-*value	Coeff.	SE	*p-*value
Individual-level variables									
Intercept	3.280	0.032	< 0.001	2.763	0.188	< 0.001	2.276	0.428	< 0.001
Gender				0.001	0.064	0.982	0.007	0.064	0.917
Age (ref = 20–39 years)									
30–59 years				0.156	0.080	0.052	0.157	0.080	0.049
60+ years				0.337	0.086	< 0.001	0.340	0.086	< 0.001
Education				0.134	0.070	0.056	0.132	0.070	0.059
Duration of residence				- 0.149	0.049	0.002	- 0.135	0.048	0.005
Living alone				- 0.276	0.073	< 0.001	- 0.274	0.071	< 0.001
Perceived financial status				0.215	0.064	< 0.001	0.214	0.064	< 0.001
Recognition of programs				0.126	0.055	0.023	0.113	0.054	0.038
Community-level variables									
Duration of programs							0.045	0.017	0.019
Number of programs							- 0.007	0.033	0.837
Recognition of program	(mean)						0.367	0.374	0.339
Random Effect	Variance Component	*χ* ^*2*^	*p-*value	Variance Component	*χ* ^*2*^	*p*-value	Variance Component	*χ* ^*2*^	*p-*value
Intercept	0.0026	22.955	> 0.500	0.0010	20.517	> 0.500	0.0003	16.948	> 0.500

*SE* Standard error, *Coeff* Coefficient, *Ref* Reference

**Table 4:** Multilevel models predicting neighborhood norm

	Model 1			Model 2			Model 3		
	Coeff.	SE	*p-*value	Coeff.	SE	*p-*value	Coeff.	SE	*p-*value
Individual-level variables									
Intercept	3.203	0.030	< 0.001	2.704	0.163	< 0.001	1.875	0.389	< 0.001
Gender				- 0.004	0.056	0.940	- 0.001	0.057	0.998
Age (ref = 20–39 years)									
40–59 years				0.187	0.084	0.027	0.191	0.084	0.023
60+ years				0.264	0.085	0.002	0.267	0.086	0.002
Education				0.135	0.066	0.042	0.148	0.066	0.026
Duration of residence				- 0.041	0.056	0.467	- 0.035	0.055	0.521
Living alone				- 0.216	0.086	0.012	- 0.207	0.086	0.016
Perceived financial status				0.114	0.072	0.113	0.117	0.071	0.099
Recognition of programs				0.112	0.046	0.015	0.089	0.047	0.058
Community-level variables									
Duration of programs							- 0.006	0.021	0.780
Number of programs							0.015	0.027	0.583
Recognition of program	(mean)						0.683	0.336	0.056
Random Effect	Variance Component	*χ* ^*2*^	*p-*value	Variance Component	*χ* ^*2*^	*p-*value	Variance Component	*χ* ^*2*^	*p-*value
Intercept	0.0008	25.548	0.322	0.0004	24.698	0.366	0.0002	19.527	> 0.500

*SE* Standard error, *Coeff* Coefficient, *Ref* Reference

As for the neighborhood norm, none of the community-level indicators proved to be statistically significant. At the individual level, living alone was negatively associated with neighborhood norm, while education was positively associated with neighborhood norm. More specifically, people between the ages of 30 and 59 and people over 60 were more positively associated with neighborhood norm than those between the ages of 20 and 39. The fit of model 3 proved better than that of the previous model.

## Discussion

This study investigates the relationship between the degree to which REPRINTS take root in the community and social capital. Our results show that not only the recognition of the REPRINTS program but also the duration of the intergenerational program is related to neighborhood trust. More specifically, people over the age of 60 and between the ages of 40 and 59 are more likely to have a positive influence on neighborhood trust than those between the ages of 20 and 39 years.

According to a national survey in Japan, older people tend to have stronger neighborhood ties than young people [44]. In addition, middle-aged people often benefit from intergenerational programs, since these programs may provide childcare or other support services. Fujiwara et al. have examined how the parents of school children participating in REPRINTS, the Japanese program studied in this paper, have a more favorable evaluation of the program after two years [45]. They conclude that REPRINTS can help establish trust between older people and the parents of school children. In contrast, younger generations are often less tied to local communities. They often move from one community to another to attend the best schools, find a job, and get married. Indeed, a 2015 demographic survey of Kawasaki found that among people in Tama Ward, those in their 20s and 30s were the most likely to move from Tama Ward to another place [46]. This study’s findings reflect the fact that older and middle-aged people tend to stay in residence longer than the younger generation. Taking all of these findings into account indicates that middle-aged and older residents are more likely to have contacts with or benefit from REPRINTS than members of the younger generation, who are newer to the area. Thus, REPRINTS seems to enhance the neighborhood trust among older and middle-aged people who have stronger neighborhood ties in a community.

Intergenerational programs like REPRINTS can be understood as bridging the social capital or social ties of heterogeneous groups. Polson et al. [47] suggest that bridging social capital is valuable to communities, since it connects individuals and leaders, inspiring them to work together for their community. To form these social networks, we assume that trust is the most important factor for social capital, since it is based on intimate familiarity with others [48]. Therefore, networking among local residents through the REPRINTS program should promote neighborhood trust. REPRINTS started several years ago in Tama Ward in Kawasaki City, and has become a well-established childcare policy over the past ten years. Murayama et al. [30] show that REPRINTS’ intergenerational exchange between senior volunteers and elementary school students positively affects students’ attitudes toward the community when they reach junior high. This finding suggests that ongoing REPRINTS programming enhances neighborhood trust among middle-aged and older local residents.

Our results support the theory that these intervention programs, while only involving senior citizens and young children, often have a spillover effect on other generations [12]. This suggests that intervention programs enhance social capital in two ways: they benefit children and senior citizens through the interventions themselves, and the community benefits through the presence of a long-term REPRINTS program.

Kaplan [49] argues that intergenerational programs providing intensive contact between generations and ongoing opportunities for intimate intergenerational engagement provide a valuable framework with which to conceptualize, categorize, and understand the impact of intergenerational programs. Other recent studies have focused on a “circle of care,” or a circle of continued intergenerational reciprocity [50–53]. According to this concept, a sustainable community relies on reciprocal relations among local residents of all generations, which may be achieved through continuous and intimate intergenerational exchanges. However, there is little evidence that the “circle of care” occurred among REPRINTS participants in this study. Therefore, a qualitative study of participants is necessary to help determine this.

The REPRINTS program was developed through cooperation with local coordinators, senior volunteers, administrative officers, and local teachers. Its collaborative nature ensures its longevity and enables it to create neighborhood trust among local residents of many generations. As such, it creates the best possible environment for positive and long-lasting benefits, enforcing social capital and building sustainable community which will reduce or prevent social isolation among older. However, programs like REPRINTS are rare, especially in Japan, where few intergenerational programs are ongoing [54]. Therefore, it is a challenge to create and implement long-term intergenerational programs that take root in local communities and boost social capital.

## Study limitations

While our study offers evidence for the relationship between social capital and the penetration of intergenerational programs, there are some limitations. First, our sample only includes active and relatively healthy adults living in a specific area. To address this concern, future studies must recruit people from varying socioeconomic conditions in a number of different communities using a random sampling technique. Second, there is a limitation in assessing neighborhood social capital with just two questions. In future research, neighborhood social capital should be evaluated using multiple standardized social capital scales. Third, this study did not consider numerous other factors that may also play a role in enhancing social capital in a community such as economy, crime rate, neighborhood beauty, cost of living, and civic engagement. Future analyses should control for these predictors. Fourth, with respect to data analysis, the results were correlational in nature; therefore, we cannot infer causality between the variables. Future studies should be conducted using panel surveys. Finally, we suggest examining differences across communities as the logical next step in this avenue of research.

## Conclusions

Overall, the findings of this study suggest that ongoing intergenerational programs between older citizens and children can reinforce neighborhood trust among local residents, thereby strengthening a community’s intergenerational ties. Moreover, this study indicates that program duration may reflect levels of community social capital. Future research is needed to show the causality between ongoing intergenerational programs and neighborhood trust, clarifying the factors that promote and inhibit the development of reciprocal relations among local residents.
